# Characteristics and quality of rotation-specific resident learning goals: a prospective study

**DOI:** 10.1080/10872981.2020.1714198

**Published:** 2020-01-15

**Authors:** Adam P. Sawatsky, Andrew J. Halvorsen, Paul R. Daniels, Sara L. Bonnes, Meltiady Issa, John T. Ratelle, Christopher R. Stephenson, Thomas J. Beckman

**Affiliations:** a Division of General Internal Medicine, Mayo Clinic, Rochester, MN, USA; b Internal Medicine Residency Program, Mayo Clinic, Rochester, MN, USA; c Division of Hospital Internal Medicine, Mayo Clinic, Rochester, MN, USA

**Keywords:** General internal medicine, graduate medical education, learning goals, learning objectives, self-directed learning, self-regulated learning

## Abstract

**Background**: Residents are expected to develop the skills to set learning goals. Setting learning goals is part of self-regulated learning, setting the foundation for creating a learning plan, deploying learning strategies, and assessing their progress to those goals. While effective goal setting is essential to resident self-regulated learning, residents struggle with setting learning goals and desire faculty assistance with goal setting.

**Objective**: We aimed to characterize the topics and quality of residents’ rotation-specific learning goals.

**Design**: We conducted a prospective study of 153 internal medicine residents, assessing 455 learning goals for general medicine inpatient rotations. We coded learning goal themes, competencies, and learning domains, and assessed quality using the validated Learning Goal Scoring Rubric. We compared topic categories, competencies, learning domains, and quality between the first and second months of postgraduate (PGY)-1 residents and between PGY-1 and PGY-3 residents. We assessed factors associated with learning goal completion.

**Results**: The overall response rate was 80%. The top three learning goal categories were patient management, specific diseases related to general medicine, and teaching skills. There were no changes in learning goal characteristics between PGY-1 months (p ≥ 0.04). There were differences between PGY-1 and PGY-3 residents’ learning goals in patient management (28% vs 6%; p < .001), specific disease conditions (19% vs 3%; p < .001), and teaching skills (2% vs 56%; p < .001). There was no difference in learning goal quality between PGY-1 months (1.63 vs. 1.67; p = 0.82). The PGY-3 learning goals were of higher quality than PGY-1 learning goals for the ‘specific goal’ item (1.38 vs. 0.98, p = 0.005), but not for other items or overall (all p ≥ 0.02). Residents reported 85% (297/347) learning goal completion.

**Conclusions**: Resident rotation-specific learning goals reflect a broad array of topics. Residents’ learning goal quality was low and residents may benefit from guidance to support residents’ learning goals.

## Introduction

The Accreditation Council for Graduate Medical Education (ACGME) Core Program Requirements highlight the importance of setting learning goals during residency training: ‘Residents must demonstrate competence in … setting learning and improvement goals’ [1]. Setting learning goals is an essential step in self-regulated learning, which Zimmerman defined as ‘self-generated thoughts, feelings and actions to attain learning goals’ and has several features, including 1) the purposive use of specific processes, strategies, or responses to improve academic achievement; 2) a self-oriented feedback loop during learning to monitor the effectiveness of the learning process, strategy, or response; and 3) a motivational dimension describing how and why a particular process, strategy, or response was chosen [2]. In clinical training in the health professions, self-regulated learning is a cyclical process, situated in a learning environment, where learners respond to clinical demands by setting goals, creating a learning plan, deploying learning strategies, and assessing their progress to those goals [3,4]. Setting effective goals affects learners’ choice of activities and increases motivation for learning, therefore increasing effort, persistence on task, and feelings of satisfaction in achieving outcomes [5].

While effective goal setting is essential to resident self-regulated learning, setting learning goals in clinical practice can be challenging. First, there are often tensions between the desired outcomes of clinical learning activities: the care of patients, the professional development of learners, and the provision of evaluations to learners and residency programs [6]. Second, residents and faculty members variably integrate learning goals into clinical learning, providing variable accountability and motivation to achieve learning goals [6,7]. Third, residents struggle with goal setting and identify the need for faculty guidance in self-regulated learning, including assistance for setting learning goals [8,9].

Research has revealed several advantageous properties of learning goals, including goal specificity and quality [5]. In order to quantify learning goal quality, Lockspeiser and colleagues developed the Learning Goal Scoring Rubric (LGSR), which assesses four items: specific goal, important goal, realistic multisource plan, and measurable outcome [10]. The LGSR has validity evidence of content and internal structure, including inter-rater reliability (ICC 0.69–0.74) [10]. Validation of the LGSR occurred in the setting of a pediatric residency program that required goal setting as part of individualized learning plans on a yearly basis, and that provided specific training to residents on setting goals. While yearly long-term goals are important, research suggests the importance of goal proximity [5] and residents have identified the potential value of setting more frequent goals that are directly related to their clinical rotations [7].

Given the importance of goal setting as a resident competency and the importance of goal proximity, more investigation is needed regarding residents’ learning goals in the context of specific clinical rotations. In this study, we explored the nature of rotation-specific goal setting. To improve our understanding of resident learning goals, we surveyed internal medicine residents on a general medicine inpatient rotation to 1) identify learning goal characteristics; 2) assess the learning goal quality using the LGSR; 3) determine changes in learning goal characteristics and quality during PGY-1 year; 4) compare learning goal characteristics and quality between PGY-1 and PGY-3 years; 5) assess learning goal completion; and 6) explore factors that affect learning goal completion.

## Materials and methods

We conducted a prospective mixed-methods study of residents learning goals for a general medicine inpatient rotation from 1 July 2015 through 30 June 2016. To characterize resident learning goals, we conducted a thematic analysis to identify major categories of learning goals. We assessed the quality of learning goals using the LGSR, and measured changes in LGSR scores over time. We examined whether learning goal characteristics were related to learning goal completion during the rotation. Lastly, we conducted a thematic analysis on narrative comments by residents regarding reasons for learning goal completion.

This study was reviewed by the Mayo Institutional Review Board and deemed exempt (IRB# 15–003474).

### Setting and participants

We invited participation by all categorical and preliminary postgraduate year (PGY) 1 and PGY-3 residents from the Mayo Clinic Internal Medicine Residency Program that rotated on a general medicine inpatient rotation during the study time frame, including 72 PGY-1 residents (48 categorical, 24 preliminary) and 48 PGY-3 residents. Residents were invited by email and participation was voluntary. The general medicine inpatient rotation was 4-weeks long and each team included one PGY-3 resident and three PGY-1 residents. Each PGY-1 resident rotated on the service twice throughout their PGY-1 year and was surveyed twice. In total, this would provide up to 144 surveys (12/month) and 432 learning goals. Each PGY-3 resident rotated on the service once; in total, this would provide up to 48 surveys (4/month) and 144 learning goals.

### Data collection

Before each general medicine inpatient rotation, residents received an email from the residency program that provided orientation to the rotation, including procedures and expectations. To create unique identifiers and de-identify the data, residents received a separate email from the primary investigator with a link to a REDCap survey, asking each resident to identify three learning goals through open-ended responses. Residents were provided the following instructions:

A learning goal is a statement that describes what you expect to achieve, including knowledge, skills and attitudes, in this case by the end of your general medicine inpatient rotation. In this survey, you will be asked to identify 3 learning goals for your current general medicine inpatient rotation. Please take some time and reflect on your learning goals prior to answering this survey.

At the end of the month-long rotation, we sent a second survey to all residents who completed the pre-rotation survey, asking the resident to recall their previously identified learning goals through open response and asking them if each learning goal had been met (yes/no). If the answer was ‘yes,’ then the resident was prompted with an open-ended question, ‘How was that learning goal met?’ If the answer was ‘no,’ then the resident was prompted with an open-ended question, ‘What were the barriers to meeting that learning goal?’

We also collected basic demographic information including postgraduate level and month of rotation.

### Data analysis

To characterize learning goals, each goal was reviewed by study team members who assigned the following characteristics: 1) learning goal theme; 2) related ACGME core competency (medical knowledge, patient care and procedural skills, practice-based learning and improvement, interpersonal and communication skills, professionalism, and systems-based practice); and 3) educational domain (knowledge, skill, or attitude). For the theme, competency, and domain, each learning goal was coded independently by two-team members, with all discrepancies reviewed and reconciled by a third team member (AS). All learning goals were linked to a unique identifier and study team members were blinded to the PGY level and timing of the rotation. One author (AS) reviewed the learning goal themes and grouped them into topic categories. These categories were then reviewed by all authors for final approval. One author (AS) used open-coding to process the qualitative comments regarding barriers or facilitators to learning goal achievement; from these codes, major themes were identified regarding the main barriers and facilitators. The thematic structure was reviewed and approved by the whole research team.

To assess the quality of learning goals, study team members applied a standardized LGSR [9]. The LGSR contains 4 items (specific goal, important goal, realistic multisource plan, and measurable outcome) each scored from 0 to 3; the total score range for the LGSR is 0–12 [9]. To train each author in the administration of this scoring tool, seven authors (AS, PD, SB, MI, JR, CS, TB) independently scored three sets of learning goals (n = 29) as a calibration exercise, iteratively reviewing our scores as an entire team and calibrating our assessment after each set of learning goals. The overall ICC for all raters was 0.922, suggesting high inter-rater reliability.

For learning category and theme, we used descriptive statistics to report the number and frequency of each category and theme. To compare learning goal category, competency, and domain between the first and second rotations for PGY-1s, and between PGY-1 and PGY-3 rotations, we used logistic regression, accounting for correlated data within residents using an exchangeable covariance structure, and appraised via Generalized Estimating Equations (GEE). For learning goal quality, mean overall and individual item scores per resident for the LGSR were compared between the first and second rotation of PGY-1 year using paired t-tests; to compare between PGY-1 and PGY-3 rotations, we used independent two-sample t-tests. To determine factors associated with learning goal completion, we used logistic regression, accounting for correlated data within residents using an exchangeable covariance structure, and assessed via GEE. To adjust for multiple comparisons, we used a more conservative (α = .01) threshold to determine statistical significance.

## Results

For the pre-rotation survey, the overall response rate was 80% (153/192); first month PGY-1 response rate was 88% (63/72), second month PGY-1 response rate was 75% (54/72), and PGY-3 response rate was 75% (36/48). For the post-rotation survey, the overall response rate was 80% (122/153); first month PGY-1 response rate was 83% (52/63), second month PGY-1 response rate was 80% (43/54), and PGY-3 response rate was 75% (27/36). A total of 455 individual learning goals identified by 105 residents were included in subsequent analyses (4 learning goals were left blank). Residents had a mean (SD) age of 28 (3) years and were 39% (N = 41) female.

### Learning goal characteristics

Residents identified a broad array of themes for their learning goals (Table 1). The main categories of these themes were patient management (103, 23%), specific disease conditions (69, 15%), teaching skills (68, 15%), efficiency (58, 13%), communication skills (41, 9%), systems-based practice (33, 7%), learning and independence (29, 6%), general medical knowledge (26, 6%), professionalism (12, 3%), physical exam skills (9, 2%), and procedural skills (7, 2%).

**Table 1.** Themes of 455 resident learning goals for a general medicine inpatient rotation

Category	Theme	No. of Learning Goals (N = 455)
Patient Management		103 (23%)
	Patient Management/Generic	20
	Diagnostic Reasoning	19
	Developing Management Plans	15
	Making Clinical Decisions	11
	Manage Acutely Ill Patients	7
	Medication Management	6
	Patient Assessment	6
	Manage Complex Patients	4
	Whole Picture/Synthesize Information	4
	Electronic Health Records	3
	Consultations	2
	ECG Reading	2
	Art of Medicine	1
	Imaging Interpretation	1
	Billing/Coding	1
	Writing Orders	1
Specific Disease Conditions		69 (15%)
	Infectious Diseases/Antibiotics	13
	Pain Management	10
	Diabetes	9
	Electrolytes/Fluid Balance/Acid-Base Disorders	9
	Acute Kidney Injury	7
	Palliative Care/Symptomatic Management	4
	Anticoagulation	3
	COPD Exacerbation	2
	Sepsis	2
	Abdominal Pain	1
	Acute Coronary Syndrome	1
	Arrhythmias	1
	Congestive Heart Failure	1
	Delirium	1
	Geriatrics	1
	Hypertension	1
	Liver disease	1
	Pneumonia	1
	Wound Care	1
Teaching Skills		68 (15%)
	Teaching Skills/Generic	24
	Case-Based Teaching	14
	Giving Feedback	10
	Learner Assessment	7
	Leading Rounds	5
	Small Group Teaching	3
	Bedside Teaching	2
	Didactic Teaching	1
	Learning Environment	1
	Mentoring	1
Efficiency		58 (13%)
	Efficiency/Generic	19
	Documentation	12
	Rounds	12
	Pre-rounds	6
	Admissions	5
	Patient Care	4
Communication Skills		41 (9%)
	Communication with Patients and Families	15
	Presentation Skills	12
	Team Management/Leadership	7
	Interprofessional Communication	5
	Communication Skills/Generic	2
Systems-Based Practice		33 (7%)
	Transitions of Care	13
	Handoffs	7
	High Value Care	7
	Systems-Based Practice/Generic	5
	Multidisciplinary Team	1
Learning and Independence		29 (6%)
	Reading Plan/SDL	14
	Independence/Autonomy	10
	Organization	4
	Reflection	1
General Medical Knowledge		26 (6%)
	Medical Knowledge/Generic	14
	General Internal Medicine Topics	12
Professionalism		12 (3%)
	Positive Attitude	4
	Team Player	3
	Patient-Centered Care	2
	Empathy	1
	Ownership/Responsibility	1
	Setting Boundaries	1
Physical Exam Skills		9 (2%)
	General Physical Exam Skills	6
	Cardiovascular Exam	2
	Musculoskeletal Exam	1
Procedural Skills		7 (2%)
	Procedures/Generic	3
	Ultrasound	3
	Paracentesis	1

We examined the breakdown of each learning goal by category, competency, and domain, and determined differences in these characteristics by month and PGY (Table 2). There were no significant differences within PGY-1 residents’ goals between their first and second months by category, competency, and domain (all p ≥ 0.04). There were significant differences between PGY-1 and PGY-3 residents’ goals in the categories of patient management (28% vs 6%, p < .0001), specific disease conditions (19% vs 3%, p = .0005), and teaching skills (2% vs 56%, p < .0001). There were significant differences between PGY-1 and PGY-3 residents’ goals in the competencies of patient care and procedural skills (31% vs 6%), medical knowledge (27% vs 6%), and practice-based learning and improvement (24% vs 67%) (all p < .0001). There were significant differences between PGY-1 and PGY-3 residents’ goals in the knowledge/cognitive (30% vs 11%, p = .0005) and skills/psychomotor domains (68% vs 82%, p = .009), but not the attitude/affective domain (2% vs 6%, p = .02).

**Table 2.** Theme, competency, domain, and postgraduate year of 455 resident learning goals

	N (%)				*P* value	
Learning Goal Characteristic	Overall (N = 455)	PGY-1, 1^st^ month (N = 189)	PGY-1, 2^nd^ month (N = 158)	PGY-3 (N = 108)	*1^st^ vs 2^nd^ month (N = 347)*	*PGY-1 vs 3 (N = 455)*
**Theme**						
Patient Management	103 (23%)	58 (31%)	39 (25%)	6 (6%)	.16	<.0001
Specific Disease Conditions	69 (15%)	35 (19%)	31 (20%)	3 (3%)	.95	.0005
Teaching Skills	68 (15%)	1 (1%)	6 (4%)	61 (56%)	.04	<.0001
Efficiency	58 (13%)	28 (15%)	18 (11%)	12 (11%)	.24	.52
Communication Skills	41 (9%)	20 (11%)	14 (9%)	7 (6%)	.59	.26
Systems-Based Practice	33 (7%)	14 (7%)	12 (8%)	7 (6%)	.96	.74
Learning and Independence	29 (6%)	12 (6%)	17 (11%)	0 (0%)	.11	*Non-Est*
General Medical Knowledge	26 (6%)	14 (7%)	9 (6%)	3 (3%)	.53	.14
Professionalism	12 (3%)	3 (2%)	2 (1%)	7 (6%)	.72	.02
Physical Exam Skills	9 (2%)	2 (1%)	6 (4%)	1 (1%)	.05	.38
Procedural Skills	7 (2%)	2 (1%)	4 (3%)	1 (1%)	.25	.58
**Competency**						
Patient Care and Procedural Skills	114 (25%)	60 (32%)	47 (30%)	7 (6%)	.65	<.0001
Medical Knowledge	98 (22%)	50 (26%)	42 (27%)	6 (6%)	.76	<.0001
Practice-Based Learning and Improvement	154 (34%)	41 (22%)	41 (26%)	72 (67%)	.21	<.0001
Interpersonal and Communication Skills	42 (9%)	20 (11%)	14 (9%)	8 (7%)	.59	.42
Professionalism	12 (3%)	3 (2%)	2 (1%)	7 (6%)	.72	.02
Systems-Based Practice	35 (8%)	15 (8%)	12 (8%)	8 (7%)	.90	.90
**Domain**						
Knowledge/Cognitive	117 (26%)	56 (30%)	49 (31%)	12 (11%)	.94	.0005
Skills/Psychomotor	325 (71%)	129 (68%)	107 (68%)	89 (82%)	.89	.009
Attitude/Affective	13 (3%)	4 (2%)	2 (1%)	7 (6%)	.46	.02

### Learning goal quality

Overall quality of the learning goals was low (Table 3). The mean score (SD) for PGY-1 first month goals was 1.63 (1.15) and for PGY-1 second month goals was 1.67 (1.40), with no significant differences seen in overall score or in any of the specific items (all p ≥ .35). The mean score for PGY-3 goals was 2.48 (1.56). PGY-3 goals were of significantly higher quality than PGY-1 goals for the ‘specific goal’ item (0.98 vs. 1.38, p = 0.005), with no difference in overall score or other individual item scores (all P ≥ 0.02).

**Table 3.** Quality of learning goals by postgraduate year for a general medicine inpatient rotation

	Mean (SD) Score		
Learning Goal Quality Measure	PGY-1, 1^st^ month (N = 47)	PGY-1, 2^nd^ month (N = 47)	*P* value
**Learning Goal Scoring Rubric**			
Specific Goal	0.91 (0.60)	0.88 (0.58)	0.72
Important Goal	0.09 (0.25)	0.07 (0.17)	0.67
Realistic Plan	0.23 (0.33)	0.26 (0.40)	0.71
Measureable Outcome	0.40 (0.38)	0.47 (0.52)	0.35
**Overall Score**	1.63 (1.15)	1.67 (1.40)	0.82
	PGY-1 (N = 69)	PGY-3 (N = 36)	
**Learning Goal Scoring Rubric**			
Specific Goal	0.98 (0.53)	1.38 (0.73)	0.005
Important Goal	0.08 (0.20)	0.06 (0.15)	0.42
Realistic Plan	0.24 (0.29)	0.44 (0.47)	0.02
Measureable Outcome	0.49 (0.40)	0.61 (0.48)	0.18
**Overall Score**	1.78 (1.15)	2.48 (1.56)	0.02

### Learning goal completion

Overall, residents reported 85% (297/347) learning goal completion. There were no significant associations between goal category, competency, domain, or quality and achieving learning goals (all p ≥ .01) (Table 4). There was a non-significant trend toward a negative association between goals that were met and overall quality (1.74 vs. 2.35, p = 0.04) and realistic plan (0.23 vs. 0.43, p = 0.04).

**Table 4.** Factors associated with completion of 347 resident learning goals for a general medicine inpatient rotation

	Goal Met?		
Factor	Yes	No	*P* Value
Characteristic of Learning Goal			
Topic			
Patient Management	69 (91%)	7 (9%)	-
Specific Disease Conditions	44 (86%)	7 (14%)	.42
Teaching Skills	35 (73%)	13 (27%)	.01
Efficiency	41 (93%)	3 (7%)	.66
Communication Skills	36 (100%)	0 (0%)	*Non-Est*
Systems-Based Practice	17 (85%)	3 (15%)	.44
Learning and Independence	17 (71%)	7 (29%)	.02
General Medical Knowledge	21 (88%)	3 (13%)	.66
Professionalism	9 (90%)	1 (10%)	.93
Physical Exam Skills	6 (75%)	2 (25%)	.22
Procedural Skills	2 (33%)	4 (67%)	.01
**Competency**			
Patient Care and Procedural Skills	75 (25%)	12 (24%)	.21
Medical Knowledge	66 (86%)	11 (14%)	.29
Practice-Based Learning and Improvement	93 (80%)	23 (20%)	-
Interpersonal and Communication Skills	36 (12%)	0 (0%)	*Non-Est*
Professionalism	9 (90%)	1 (10%)	.46
Systems-Based Practice	18 (86%)	3 (14%)	.54
**Domain**			
Knowledge/Cognitive	76 (84%)	14 (16%)	.77
Skills/Psychomotor	211 (86%)	35 (14%)	-
Attitude/Affective	10 (91%)	1 (9%)	.65
**Quality of Learning Goal**			
**Learning Goal Scoring Rubric**	N = 295	N = 49	
**Overall Score**	1.74 (1.77)	2.35 (2.45)	.04
Specific Goal	0.99 (0.91)	1.16 (0.96)	.99
Important Goal	0.07 (0.30)	0.14 (0.41)	.31
Realistic Plan	0.23 (0.49)	0.43 (0.68)	.04
Measureable Outcome	0.45 (0.68)	0.61 (0.91)	.84

For the learning goals that were achieved, we examined facilitators for goal completion; for goals that were not achieved, we examined barriers to goal completion. We received responses for 70% (317/455) of the goals; many responses contained more than 1 barrier or facilitator, for a total of 369 individual barriers or facilitators. There were 314 reported facilitators for goal completion; the main facilitators fell under the categories of intentional effort (128, 41%), external guidance (98, 31%), and clinical experience (88, 28%). There were 55 reported barriers to goal completion; the main barriers were time (19, 35%), lack of opportunity (16, 29%), other priorities (4, 7%), unrealistic goal (2, 4%), forgotten goal (2, 4%), and other (12, 22%).

## Discussion

In this study, we examined the characteristics and quality of rotation-specific resident learning goals for a general medicine inpatient rotation. The learning goal themes were broad and encompassed topics across the ACGME core competencies. Learning goals differed between first and third-year residents; PGY-1s’ goals focused on attaining the knowledge and skills to care for patients and PGY-3s’ goals focused on attaining team management and teaching skills. The overall quality of learning goals was low, with little difference in quality between first- and third-year residents; however, learning goal quality was not associated with learning goal completion. The majority of residents reported achieving their goals, with facilitators equally divided between clinical experience, intentional effort, and external guidance.

Previous research has shown that residents struggle with self-regulated learning, including goal setting [8,9]. Residents identified that faculty can provide guidance by helping them identify important and appropriate learning goals [9]. This study shows that residents set a variety of learning goals across ACGME core competencies and knowledge, skills, and attitudes. Yet, there may be specific ways in which faculty members can better support the formulation of learning goals. First, there were fewer goals associated with interpersonal and communication skills, professionalism, and systems-based practice than the other competencies. Second, the overall quality of learning goals in this study was low. One possible explanation for this is that within the natural setting of residency training there is not adequate time and training for learning goals. It was informative that residents reported the main facilitators to completion were intentional effort (such as self-learning) and guidance from others including faculty, other residents, or other members of the health-care team. This supports previous work suggesting that program- and faculty-level for support of self-regulated learning, including goal setting, is important and may improve learning outcomes [7,11].

While we demonstrated a high rate of learning goal completion, there was no association between goal completion and the characteristics or quality of the goal. One explanation is that we made learning goal completion a binary outcome, when in reality, self-regulated learning is a cyclical process that often leads to revision of goals and learning strategies over time [3,4]. Second, a vague or ill-defined goal may be more likely to be viewed as completed by the learner, because the criterion for completing the goal is not clear. While completing a goal may improve self-efficacy, the effect on learning is less clear. Third, residents may set goals that they are already close to completing in order to look good for their supervisors. Learners will interact with goal setting and feedback based on their understanding of the learning environment; when learners perceive goal setting and feedback as both formative and summative assessments of performance, they may set goals they are likely to complete than setting challenging goals [12]. More research is needed to clarify the relationship between goal quality, goal completion, and learning outcomes.

Recent research on goal setting in medical education can provide insight into improving the quality of learning goals in the clinical setting. Saddawi-Konefka and colleagues borrowed the concept of ‘implementation intentions’ from the cognitive psychology literature, having faculty ask learners to set goals and anticipate situations that present obstacles to goal achievement and creating ‘if-then’ plans for how to respond to these obstacles to progress toward goals [13]. They also conducted a trial of structured development of implemented intention statements (WOOP – wish, outcome, obstacle, plan) versus regular goal setting, demonstrating that residents spend more time reading toward their goal with the use of implemented intentions [14]. Therefore, providing faculty support for goal setting and contingency planning for obstacles may be better than providing support for goal setting alone. From their qualitative exploration using cultural-historical activity theory, Larsen and colleagues suggest that learning goals can serve as a tool that unites students, supervisors, and patients, and align the clinical education outcomes of professional development, grading, and patient care [6]. This alignment occurred when faculty asked learners to set goals, incorporated learners’ goals in the team’s work, and provided feedback to learners on their goals [6]. These studies suggest that increasing faculty engagement with residents’ learning goals can improve learners’ progress toward their goals.

This study provides mixed evidence for relations to variables (criterion) validity evidence for the LGSR [15]. We saw changes in the themes and competencies of resident learning goals across PGY years, suggesting a growth in learning goal setting over the course of residency training. That being said, the overall quality of learning goals did not change over time. One possible explanation is that gaining clinical experience alone does not improve the quality of residents’ learning goals. Interestingly, there was an increase in the ‘specific goal’ item for PGY-3s versus PGY-1s. This corresponds to previous work on resident self-regulated learning, where residents demonstrated, over time, an ability to develop more intricate knowledge frameworks with smaller knowledge gaps, driving the creation of more specific learning goals [4]. Another possibility is that the LGSR could be further refined for the measurement of learning goal quality. Consequently, further research with the LGSR could focus on correlations with other measures of self-directed learning and with future achievement.

This study has several limitations. First, we included trainees from a single specialty at one institution. Although this research builds upon previous studies from pediatric residency programs, more investigations are needed on validity of the LGSR in other populations. Second, this is an observational study that did not involve training on developing learning goals or any other intervention; nonetheless, characterizing the quality of learning goal development in a natural setting provides new information about resident self-regulated learning. Further research is needed to correlate the LGSR with other measures of self-regulated learning, like the Motivated Strategies for Learning Questionnaire [16] or microanalytic techniques [17]. Finally, this study does not provide evidence of correlations between the quality of learning goals and educational outcomes.

## Conclusions

We found that resident learning goals reflect a variety of topics across core graduate medical education competencies and that PGY-3 residents develop learning goals beyond medical knowledge to include more integrative abilities such as team management and teaching skills. Residents’ learning goal quality may be low without specific guidance on goal development, and there are several facilitators and barriers to goal completion. We are hopeful that these findings will help to guide faculty members and residency programs as they strive to support resident self-regulated learning and guide future research on associations between learning goal quality, goal completion, and learning outcomes.

## Data Availability

There is no data set associated with the paper.

## Prior Presentations

This study was presented at the Society of General Internal Medicine 2019 Annual Meeting on 10 May 2019, in Washington, D.C. and the abstract was published in the Journal of General Internal Medicine (Journal of General Internal Medicine. 2019; 34(supp 2):S161.)
